# Germ Cell Nuclear Factor (GCNF) Represses Oct4 Expression and Globally Modulates Gene Expression in Human Embryonic Stem (hES) Cells*[Fn FN2]

**DOI:** 10.1074/jbc.M115.694208

**Published:** 2016-01-14

**Authors:** Hongran Wang, Xiaohong Wang, Xueping Xu, Michael Kyba, Austin J. Cooney

**Affiliations:** From the ‡ Department of Pediatrics, Dell Pediatric Research Institute, University of Texas at Austin Dell Medical School, Austin, Texas 78723,; § Stem Cell Center, Texas Heart Institute, Houston, Texas 77030,; ¶ Department of Molecular and Cellular Biology, Baylor College of Medicine, Houston, Texas 77030, and; ‖ Department of Pediatrics, Lillehei Heart Institute, University of Minnesota, Minneapolis, Minnesota 55454

**Keywords:** cell differentiation, embryonic stem cell, gene expression, gene regulation, pluripotency, Germ cell nuclear factor, Oct4

## Abstract

Oct4 is considered a key transcription factor for pluripotent stem cell self-renewal. It binds to specific regions within target genes to regulate their expression and is downregulated upon induction of differentiation of pluripotent stem cells; however, the mechanisms that regulate the levels of human Oct4 expression remain poorly understood. Here we show that expression of human Oct4 is directly repressed by germ cell nuclear factor (GCNF), an orphan nuclear receptor, in hES cells. Knockdown of GCNF by siRNA resulted in maintenance of Oct4 expression during RA-induced hES cell differentiation. While overexpression of GCNF promoted repression of Oct4 expression in both undifferentiated and differentiated hES cells. The level of Oct4 repression was dependent on the level of GCNF expression in a dose-dependent manner. mRNA microarray analysis demonstrated that overexpression of GCNF globally regulates gene expression in undifferentiated and differentiated hES cells. Within the group of altered genes, GCNF down-regulated 36% of the genes, and up-regulated 64% in undifferentiated hES cells. In addition, GCNF also showed a regulatory gene pattern that is different from RA treatment during hES cell differentiation. These findings increase our understanding of the mechanisms that maintain hES cell pluripotency and regulate gene expression during the differentiation process.

## Introduction

Embryonic stem (ES)^3^ cells are derived from the inner cell mass (ICM) of pre-implantation mouse (1, 2) and human (3) blastocysts. The pluripotent properties of ES cells are maintained by several key regulatory genes (4, 5), but the molecular mechanisms controlling pluripotency, self-renewal, and cell fate decisions are not completely defined. *Oct4*, which belongs to the *POU* homeodomain gene family, is one of the key transcription factors that play a fundamental role in the maintenance of ES cell pluripotency by blocking differentiated gene expression (6, 7).

*Oct4* is precisely regulated throughout the entire embryonic and fetal developmental processes. After oocytes are fertilized, Oct4 is expressed in the blastomeres, inner cell mass (ICM), and epiblasts (8). Oct4 expression is subsequently down-regulated in somatic cells during gastrulation. At later stages of development, Oct4 is only found in primordial germ cells (9). *In vitro*, Oct4 is found in ES cells and embryonal carcinoma (EC) cells and is down-regulated when these cells are induced to differentiate with retinoic acid (RA) treatment or by removing leukemia inhibitory factor (LIF) (10, 11). These distinctive expression patterns of Oct4 during early mouse development and in undifferentiated cell lines imply that *Oct4* is regulated in a temporal-spatial manner.

Germ cell nuclear factor (GCNF), an orphan nuclear receptor, was initially described to have tissue-specific expression in germ cells of the adult mouse (12) and humans (13, 14). GCNF mediates repression of Oct4 in mouse ES cells and induced pluripotent stem (iPS) cells by binding to a DR0 response element within the *Oct4* promoter and recruiting DNA methyltransferases leading to silencing of *Oct4* expression during differentiation of mouse ES cells (15, 16). GCNF expression dramatically increases during gastrulation while Oct4 expression decreases; GCNF expression pattern of tempo-spatial variation is inversely associated with Oct4 expression during mouse embryonic development, and GCNF itself is essential for normal embryonic development (17, 18). Loss of GCNF function in GCNF knock-out mice results in embryonic lethality by embryonic day (E) E10.5, with a complex set of phenotypes leading to posterior truncation and includes defects in forebrain development, and the establishment of the isthmic organizer (17, 18, 19). Importantly, there is an overt loss of normal repression of Oct4 expression in somatic cells after gastrulation, a stage at which Oct4 is normally silenced (20).

Human embryonic stem cells are powerful tools to study early human development *in vitro*. Moreover, they provide a source of cells with therapeutic potential in regenerative medicine (21, 22). Understanding the mechanisms controlling Oct4 expression would help us regulate pluripotency and differentiation of hES cells. However, many aspects of ES cell regulation are species specific and thus it is important to know which mechanisms discovered in the mouse model translate to the human system and thereby would have clinical relevance. The high level of GCNF conservation among mammalian species suggests that GCNF probably is a candidate factor in the regulation of Oct4 expression during early human embryonic development and human ES (hES) cell differentiation. Here we report that GCNF is inducible, represses Oct4 expression during RA-induced human ES cell differentiation, and globally modulates gene expression.

## Experimental Procedures

### 

#### 

##### hES Cell Maintenance

H9 hES cells and hGCNF transfected H9 hES (G-hES) cells were cultured on feeder cells or on Matrigel (catalogue #354230, BD Biosciences) coated plates in mTeSR™1 medium (catalogue #05850, Stemcell Technologies). Medium used for growth of undifferentiated cells (UM): DMEM/F12, 20% knock-out serum replacement, 1× non-essential amino acids, 0.1 mm β-mercaptoethanol, 4 ng/ml bFGF. Medium used for growth of differentiated cells (DM): DMEM/F12, 10% FBS, 1× non-essential amino acids, 0.1 mm β-mercaptoethanol. Collagenase type IV (catalogue #17104-019, Invitrogen) was used to disperse hES cells. hES cells were passaged every 5 or 6 days.

##### Small Interfering RNA (siRNA) GCNF Knockdown

H9 hES cells were cultured on Matrigel-coated plates in mTeSR™1 medium (catalogue #05850, Stemcell Technologies). 20 nm of GCNF siRNA, siGENOME SMARTpool (catalogue #M-003431-01, Dharmacon). siCONTROL Non-Targeting siRNA Pool #1 (catalogue # D-001206-13-20, Dharmacon) was used as negative control. siRNA was transfected into cells using Lipofectamine 2000 transfection reagent according the manufacturer's instructions.

##### Plasmid Construction and Transfection

Human GCNF cDNA was obtained via PCR of human ES cell cDNA and was cloned into a gateway plasmid, which was introduced into a lentiviral parent plasmid (pSAM2-GW); the sequence between attR1 and attR2 was replaced with the hGCNF gene sequence.

##### RNA Isolation and Analysis

Total RNAs were isolated from the samples using TRizol reagent (catalogue #15596018, Invitrogen). RNA samples were analyzed with reverse transcription (RT)-PCR or quantitative real-time polymerase chain reaction (qRT-PCR) with SYBR green Q-PCR reagent (catalogue # 208056, Qiagen) and PCR primers as listed below: Human GCNF forward: CCCAGTCATACAGTCTGAT, reverse: AAGCAGGGCAAATAGTTCT; Human Oct4 forward: AATCTTCAGGAGATATGCAAAG, reverse: CTGGGCGATGTGGCTGATCT; Human Sox2 forward: CTTCACATGTCCCAGCAC, reverse: CTCCCATTTCCCTCGTTT; Human 18s forward: GAATGAGTCCACTTTAAATCCT, reverse: CAAGATCCAACTACGAGCTTTT.

##### Gene Expression Profiling and Analysis

Differential gene expression among different groups of hES cells was assayed with MCF Affymetrix HG-U133_Plus_2 GeneChips. Undifferentiated hES cells were cultured with or without Dox for 4 days; hES cells were treated with or without 1 μm RA or Dox for 6 days to induce hES cell differentiation in differentiation media, in triplicate. Differentiated hES cells were treated with RA or Dox. The Baylor College of Medicine Microarray Core Facility labeled the cRNA used in these experiments. Microarray data were analyzed with DNA Chip Analyzer software.

##### Western Blot Analysis

Total cell lysates were subjected to Western blot analyses. The following antibodies were used: primary antibodies against: Oct4 (catalogue# sc-5279, Santa Cruz Biotechnologies), GCNF (15), β-actin (catalogue #A1978, Sigma), and Sox2 (catalogue# AB5603, Thermo Scientific); secondary antibodies: goat anti-mouse IgG-HRP (catalogue# sc-2005, Santa Cruz Biotechnology), and goat anti-rabbit IgG-HRP (catalogue# sc-2030, Santa Cruz Biotechnology). HRP activity was detected with a chemiluminescent methodology using Pierce ECL Western blotting Substrate kit (Thermo Scientific). The emitted light was detected by photographic film.

##### Electrophoretic Mobility Shift Assays (EMSAs)

Nuclear extracts were prepared from undifferentiated and differentiated NT-2 cells at time points of 1, 2, 3, 4, 5, and 6 days. Oligonucleotides with a sequence complementary to the GCNF binding site were labeled with P^32^ isotope and incubated with nuclear extracts before samples were loaded for electrophoresis. GCNF antibody was added to the mixture of probe and nuclear extracts in EMSA experiments to identify specific complexes.

##### Chromatin Immunoprecipitation Analysis

Sonicated DNA samples of undifferentiated and differentiated ES cells at different time points were used for chromatin immunoprecipitation (ChIP) assays. The antibodies that were used for the ChIP assays were against GCNF and normal rabbit IgG was used as a control. qRT-PCR was carried out using SYBR Green FastMix (Qiagen) with an ABI 7900HT real-time PCR machine. The following primer sets spanning the Oct4 promoter were used to test GCNF binding to the Oct4 proximal promotor DR0 element: forward: 5′-ACCTCCCTCTCCTCCACCCAT-3′; reverse: 5′-GAAGGGACTACTCAACCCCTCTCT-3′. The primer sets spanning the Oct4 genomic intron 1 were used as a control to test for nonspecific binding of GCNF as following: forward: 5′-AGTCCAAAGTCTGGTCCCTTGAA-3′; reverse: 5′-TCCAGAATCAGACTCCAGACTCTCCT-3′.

##### Statistical Analysis

All data were obtained from triplicate experiments and presented as mean ± S.D. Student's *t* test was performed to determine the differences among grouped data. * indicates statistically no significance with *p* ≥ 0.05; ** indicates statistically significance with *p* < 0.05.

## Results

### 

#### 

##### GCNF Binding to the DR0 Element within the Oct4 Promotor in Human Cells

**FIGURE 1.**
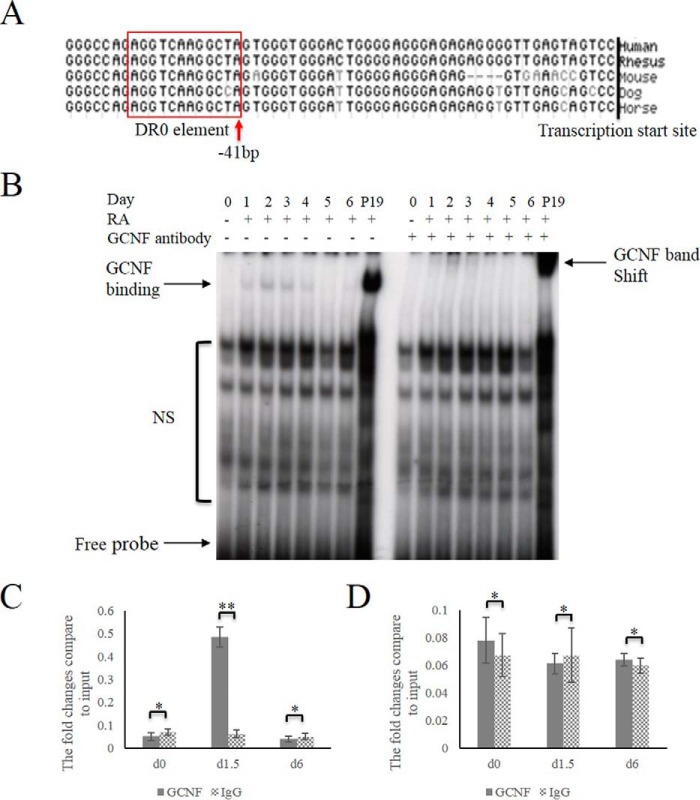
**GCNF binding DR0 element in human cells.** *A*, A DR0 sequence is located within the Oct4 promoter of different species. *B*, EMSA was performed to test GCNF binding to the DR0 element within the Oct4 promoter. P19 cell extracts were used as a positive control. *NS*: nonspecific binding. *C*, qRT-PCR results of GCNF binding the DR0 element within the Oct4 promotor. *D*, qRT-PCR results of GCNF binding control DNA sequence within intron 1 of Oct4 genome. * indicates no statistically significance with *p* ≥ 0.05; ** indicates statistically significance with *p* < 0.05.

Our previous studies showed that GCNF represses and silences *Oct4* by binding to the DR0 sequence in mES cells. Comparison of the promoter of Oct4 among different species, identified a conserved DR0 element AGGTCAAGGCT(C)A located within the proximal promoter of the Oct4 gene not only in human and mouse but also in other species analyzed (Fig. 1*A*). GCNF itself is also highly conserved among species. The human cDNA encoding GCNF is 98.7% identical to the equivalent cDNA encoding the mouse protein (13, 14) and has identical DNA-binding domains (14, 23). Therefore, we hypothesized that hGCNF also regulates *Oct4* in human cells. In order to test if GCNF binds the DR0 element located within the *Oct4* promotor in human cells, electrophoretic mobility shift assay (EMSA) was used in experiments. The results showed that a probe containing the *Oct4* DR0 element formed retarded complexes with nuclear extracts from human embryocarcinoma cells on day 1 of RA induced differentiation. The shifted bands were further retarded with anti-GCNF antibodies, which is consistent with the results derived from the positive control mouse P19 cell nuclear extracts (Fig. 1*B*), demonstrating that hGCNF can bind to the *hOct4* promotor.

To further analyze GCNF binding to the Oct4 promoter *in vivo*, ChIP was performed at day 0 (undifferentiated) and at days 1.5 and 6 of RA induced differentiation. The results showed that GCNF bound the DR0 element at a high level at days 1.5 of differentiation (Fig. 1*C*). In the control, GCNF did not bind the Oct4 intron 1 DNA (Fig. 1*D*).

##### GCNF Is Necessary for Inhibition of Oct4 during hES Cell Differentiation

**FIGURE 2.**
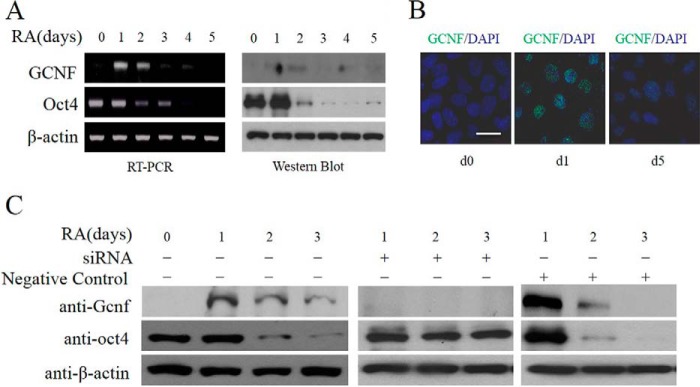
**GCNF expression during hES cell differentiation.** *A*, PCR and Western blot results of GCNF and Oct4 expression in undifferentiated and differentiated hES cells. *B*, immunostaining of GCNF in undifferentiated hES cells (d0) and differentiated hES cells (d1 and d5) treated with RA. *C*, siRNA-mediated inhibition of GCNF during hES cell differentiation; Western blot results of GCNF and Oct4 expression; β-actin was used as a loading control. *Left panel*: samples without treatment; *middle panel*: samples with GCNF siRNA treatment; *right panel*: negative control (*NC*) with non-targeting siRNA treatment. Scale bar: 20 μm.

To further investigate the effects of GCNF on the regulation of the *Oct4* gene in human pluripotent cells, RA was used to induce hES cell differentiation. During differentiation, GCNF expression was induced from day 1 of differentiation (d1) onwards and subsequently its expression gradually decreased. Results of Western blot and RT-PCR analyses (Fig. 2*A*) showed that, in response to increased GCNF, Oct4 expression decreased rapidly during differentiation. Immunostaining experiments with anti-GCNF antibody also verified that GCNF was transiently induced during RA-induced differentiation, it was expressed within the cell nucleus at day 1 of differentiation, and decreased by day 5 of differentiation (Fig. 2*B*).

To validate whether inhibition of *GCNF* led to loss of repression of Oct4 expression during hES differentiation, small interfering RNA (siRNA) (7) were used to inhibit GCNF expression during RA-induced differentiation. Oct4 expression was maintained after GCNF expression was knocked down by siRNA, while the expression level of Oct4 decreased rapidly in control cells. These results showed that GCNF is necessary for inhibition of Oct4 expression during hES cell differentiation (Fig. 2*C*).

##### GCNF Overexpression Repressed Oct4 in Undifferentiated hES Cells

**FIGURE 3.**
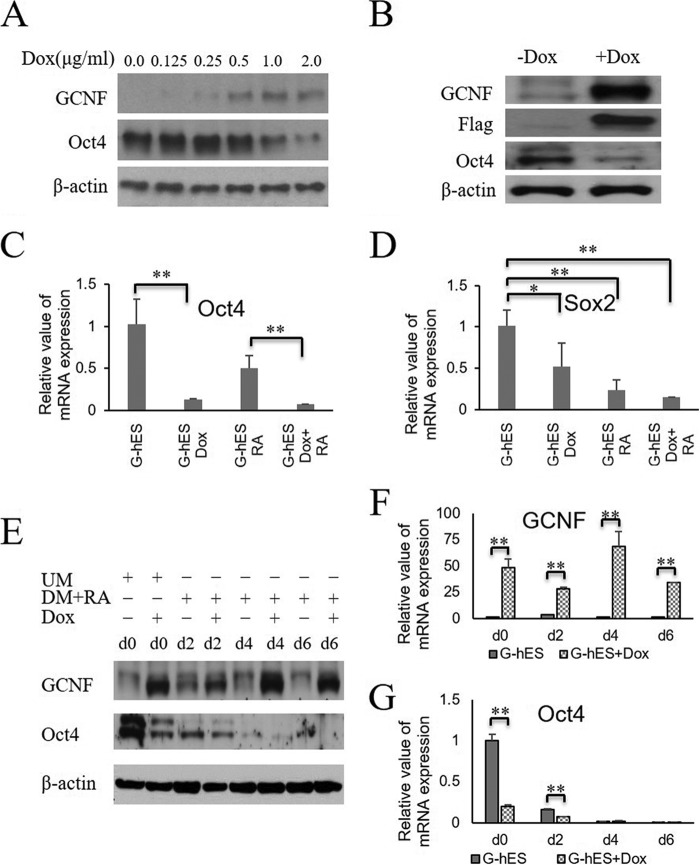
**Overexpression of GCNF inhibits Oct4 expression.** *A*, Western blot results of the level of GCNF and Oct4 proteins in undifferentiated G-hES cells treated with Dox (0.0, 0.125, 0.25, 0.5, 1.0, 2.0 μg/ml) for 4 days. β-Actin was used as a loading control. *B*, Western blot results of GCNF, Flag tag GCNF, Oct4, and β-actin expression in undifferentiated G-hES cell treated with Dox and without Dox for 4 days. *C*, Oct4 mRNAs were detected in undifferentiated G-hES cells, Dox-treated G-hES cells, RA-treated G-hES cells, and Dox-plus-RA treated G-hES cells. *D*, Sox2 mRNAs were detected in undifferentiated G-hES cells, Dox-treated G-hES cells, RA-treated G-hES cells, and Dox-plus-RA treated G-hES cells. *E*, Western blot results of GCNF and Oct4 expression in undifferentiated G-hES treated with Dox for 4 days, and in differentiated G-hES cells treated with RA or plus Dox for 2, 4, and 6 days. *F*, qRT-PCR results of GCNF expression in undifferentiated G-hES treated with Dox for 4 days and in differentiated G-hES cells treated with RA or plus Dox for 2, 4, and 6 days. *G*, qRT-PCR results of Oct4 mRNA expression in undifferentiated G-hES treated with Dox for 4 days and in differentiated G-hES cells treated with RA or plus Dox for 2, 4, and 6 days. * indicates no statistically significance with *p* ≥ 0.05; ** indicates statistically significance with *p* < 0.05.

GCNF is expressed at very low levels in undifferentiated hES cells, at a level insufficient to repress and silence Oct4 expression. To examine whether GCNF can inhibit Oct4 expression in undifferentiated hES, we constructed a human GCNF-Flag-IRES-GFP lentiviral vector with the doxycyclin (Dox) inducible system, and introduced it into H9 hES cells. To induce GCNF expression, transfected hES (G-hES) cells were treated with different concentrations (0.0, 0.125, 0.25, 0.5, 1.0, 2.0 μg/ml) of Dox for 2 days. After the induction, GCNF expression was validated by Western blots. The results showed that Dox induced GCNF overexpression in a dose-dependent manner and that decreasing levels of Oct4 were negatively correlated with GCNF expression. 1.0 μg/ml of Dox was sufficient to induce both GCNF expression and significant inhibition of Oct4 (Fig. 3*A*), thus 1.0 μg/ml of Dox was used in the following experiments.

To exclude the influence of Dox on Oct4 expression, and validate that reduction of Oct4 expression was caused by the expression of GCNF itself, we treated non GCNF-transfected H9 ES cells with 1.0 μg/ml of Dox for 4 days. The levels of GCNF, Oct4, and Sox2 were not significantly affected when compared with the corresponding levels found in H9 hES cells in non-Dox-treatment controls by using quantitative real-time polymerase chain reaction (qRT-PCR) verification (data not shown). However, GCNF was induced and Oct4 was inhibited at day 4 in undifferentiated G-hES cells (Fig. 3*B*). Thus, the reduction of Oct4 levels was induced by the increase of GCNF overexpression in undifferentiated G-hES cells. To further confirm that Dox can induce recombinant GCNF, it was indirectly verified by detecting the fused-protein Flag tag, which is co-expressed with GCNF in the human GCNF-Flag-IRES-GFP lentiviral vector. Western blot results for the Flag tag showed a band corresponding to that of GCNF (Fig. 3*B*). These results are the first gain-of-function experiments that show that GCNF overexpression inhibited Oct4 expression in undifferentiated hES cells.

To compare the effects between RA and GCNF on Oct4 expression in undifferentiated G-hES cells, the cells were treated with Dox, RA, or Dox plus RA for 4 days, respectively. Results showed that GCNF expression was induced by either Dox, or Dox plus RA treatment; Oct4 expression was repressed about 80% more compared with RA treatment alone; and RA enhanced the repression of Oct4 in undifferentiated hES cells (Fig. 3*C*). Surprisingly, the expression level of Sox2 did not change as much as the expression of Oct4 in undifferentiated cells treated with Dox, likely because Sox2 is not a direct target of GCNF, consistent with the absence of a DR0 element anywhere near the Sox2 gene. However, treatment with RA or Dox plus RA resulted in significant repression of Sox2 expression (Fig. 3*D*).

To further validate synergistic effects between GCNF and RA on enhancing reduction in Oct4 expression during hES cell differentiation, G-hES cells were treated with RA alone or with both RA and Dox. Undifferentiated G-hES cells treated with or without Dox for 4 days were used as controls, respectively. Western blot and qRT-PCR experiments showed that Dox induced overexpression of GCNF in undifferentiated and differentiated G-hES cells (Fig. 3, *E* and *F*); the level of Oct4 decreased considerably more in cells treated with RA plus Dox than in cells treated with RA alone. (Fig. 3, *E* and *G*). Greater than 50% inhibition of Oct4 expression was elicited by overexpression of GCNF in G-hES cells at day 2 of differentiation, which is similar to the inhibition of Oct4 observed at day 4 in Dox-treated undifferentiated G-hES cells. At later stages of differentiation, Oct4 was almost completely repressed at days 4 and 6 in cells treated with or without Dox (Fig. 3, *E* and *G*). These data indicate that increased levels of *GCNF* expression can enhance the reduction of Oct4 expression during hES cell differentiation.

##### GCNF Is Sufficient to Induce Differentiation of hES Cells

**FIGURE 4.**
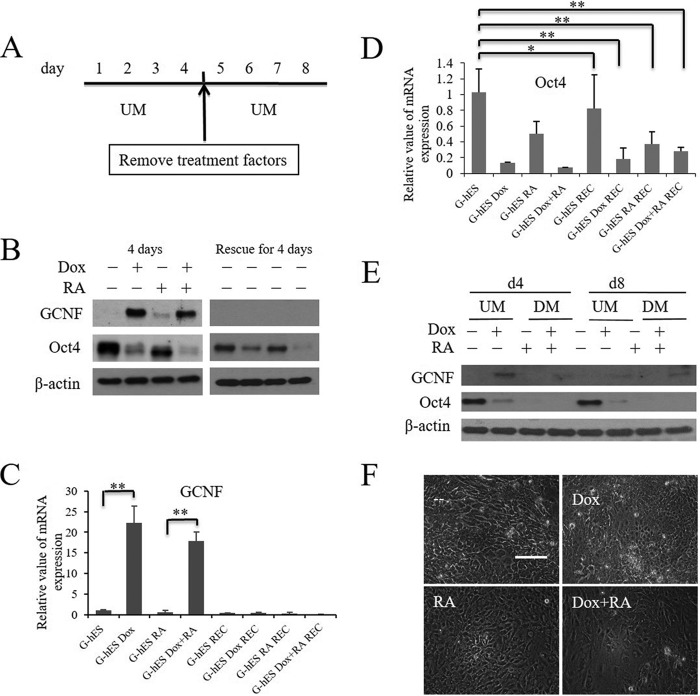
**Overexpression of GCNF induced the differentiation of hES cells.** *A*, rescue experiment diagram: G-hES cells were treated with Dox, RA, and Dox plus RA for 4 days, and then cultured without treatment factors for another 4 days. *B*, Western blot results of GCNF and Oct4 expression in G-hES cell cultures treated with or without Dox, RA, and Dox plus RA for 4 days, and expression after those treatments were removed for another 4 days. *C*, analysis of qRT-PCR results of GCNF expression in rescue experiments. *D*, analysis of qRT-PCR results of Oct4 expression in rescue experiment. *E*, Western blot results of GCNF and Oct4 expression in G-hES cell cultures at day 4 or day 8 in UM or DM that included RA or RA plus Dox. *F*, morphological changes after G-hES cells were treated with Dox, RA, and Dox plus RA for 8 days. Untreated G-hES cells were used as control. Scale bar: 50 μm. *UM* stands for medium used for growth of undifferentiated cells; *DM* stands for medium used for growth of differentiated cells. * indicates no statistical significance with *p* ≥ 0.05; ** indicates statistical significance with *p* < 0.05.

Based on the repression of *Oct4* by GCNF overexpression in undifferentiated hES cells and that Oct4 is a key transcription factor for maintaining the pluripotent properties of hES cells, our question was whether GCNF expression was sufficient to initiate differentiation of hES cells. Thus, we performed an Oct4 rescue experiment to determine whether the inhibition of Oct4 could be recovered after Dox was withdrawn. The cells were treated as described in Fig. 4*A*. G-hES cells were cultured in UM with Dox, RA, or Dox plus RA for 4 days, and then the cells were maintained in UM without RA or Dox treatment for a further 4 days. Untreated G-hES cells were used as controls. GCNF expression was lost after removal of Dox; Oct4 was inhibited after Dox, RA, or Dox plus RA treatment for 4 days (Fig. 4*B*). The expression of GCNF and Oct4 mRNAs was similar to the corresponding protein levels (Fig. 4, *C* and *D*). Analysis of Western blot (Fig. 4*B*) and qRT-PCR (Fig. 4*D*) results showed that after being rescued for 4 days, Oct4 levels were not restored to the levels present in ES cells prior to treatment.

After treatment with RA or Dox plus RA, cells lost their typical morphological characteristics of undifferentiated hES cells and changed into fibroblast-like cells. No morphological changes were observed after treating the cells with Dox for 4 days (data not shown). Cell morphology still did not change after the cells were rescued for 4 days with Dox treatment. However, differentiated cell morphologies were observed in RA or Dox plus RA groups although treatment had been removed (data not shown). These results indicated that GCNF inhibited Oct4 expression triggering hES cell differentiation.

To further validate the ability of GCNF to induce hES cell differentiation, we extended the Dox treatment time. After 8 days of Dox treatment, the expression of the Oct4 gene was repressed and almost silenced under undifferentiated culture conditions on feeder cells (Fig. 4*E*). In comparison, Oct4 completely disappeared by day 4 or day 8 in differentiation media that included RA or RA plus Dox (Fig. 4*E*). After 8 days of Dox treatment, cells adopted a differentiated morphology: becoming bigger and the ratio of nucleus to cytoplasm increased, similar to the cells treated with RA or Dox plus RA (Fig. 4*F*). These results indicated that GCNF expression is sufficient to induce differentiation of hES cells even when the cells are cultured on feeder cells in UM.

##### GCNF Modulated Global Gene Expression in Undifferentiated and During Differentiation of hES Cells

**FIGURE 5.**
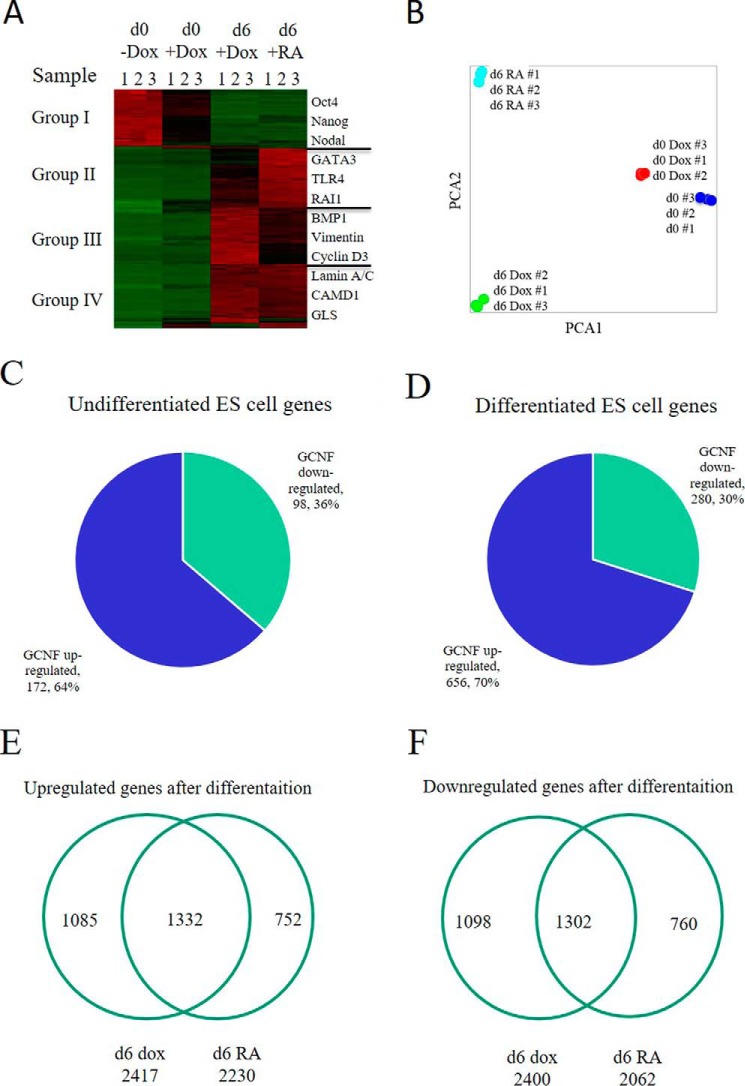
**GCNF regulates gene expression in both undifferentiated and differentiated hES cells by mRNA array analysis.** *A*, RNA microarray analysis of undifferentiated (d0) G-hES cells, d0 G-hES cells with Dox treatment, differentiated (d6) G-hES cells with Dox treatment, and d6 G-hES cells with RA treatment. The level of expression of mRNA was divided into four groups: *Group I*: genes that were expressed at high levels in d0 G-hES cells and whose level was very low after RA or Dox-induced differentiation. *Group II*: genes that were expressed in d6 G-hES cells at higher levels in RA-treated cells than in Dox-treated cells. *Group III*: genes that were expressed in d6 G-hES cells at higher levels in the Dox-treated cells than in RA-treated cells. *Group IV*: genes that were up-regulated in Dox-treated or in RA-treated d6 G-hES cells. Some genes whose expression markedly changed were listed beside the groups. *B*, PCA map from RNA microarray analysis of the different groups. *C*, overexpression of GCNF affected gene expression in undifferentiated hES cells. *D*, overexpression of GCNF affected gene expression during hES cell differentiation. *E*, genes up-regulated by Dox or RA treatment during G-hES cell at day 6 of G-hES cell differentiation. *F*, genes down-regulated by Dox or RA treatment at day 6 of G-hES cell differentiation.

We then asked how important GCNF was to repression of the entire pluripotent state during ES cell differentiation and whether other genes were also affected by GCNF overexpression. To examine this issue, RNAs were collected from undifferentiated G-hES cells, Dox-treated undifferentiated G-hES cells, differentiated G-hES cells at day 6 of Dox treatment, and differentiated G-hES cells at day 6 of RA treatment. We analyzed changes in gene expression patterns in RNA samples using microarray technology. Microarray data showed a ≥2-fold change in gene expression, and a difference in the level of gene expression >100 between the two groups (*p* ≤ 0.05). The heat map showed that GCNF overexpression down-regulated to some extent genes highly expressed in undifferentiated hES cells. We observed those genes to be completely repressed after RA-induced hES cell differentiation (group I), for example, Oct4, Nanog and Nodal (Fig. 5*A*). The rest of the genes, which were expressed at very low levels in undifferentiated hES cells, were up-regulated in differentiated hES cells treated with RA or Dox (groups II-IV) (Fig. 5*A*). The results of the Principal Component Analysis (PCA) of the full microarray clearly illustrated these trends. The PCA map also showed that the gene expression pattern is different between undifferentiated hES treated with Dox and the cells without Dox treatment. The differences in gene expression were also found at day 6 between differentiated hES cells treated with RA and those treated with Dox (Fig. 5, *A* and *B*).

Gene expression was also compared between undifferentiated ES cells treated with or without Dox. Overexpression of GCNF led to down-regulation of 36% (98) and up-regulation of 64% (172) of the total differentially expressed genes in G-hES cells when they were treated with Dox for 4 days (Fig. 5*C*). Using the same methodology to analyze gene expression between Dox-treated and RA-treated differentiated ES cells for 6 days, the results showed that the expression of 936 genes was significantly different between undifferentiated ES cells treated with or without Dox. Globally 30% (280) of the genes were downregulated and 70% (656) genes were up-regulated by Dox treatment compared with RA treatment (Fig. 5*D*).

The difference in regulating global gene expression was compared between RA and Dox treatment in undifferentiated and differentiated G-hES cells. Compared with undifferentiated G-hES cells, RA-treated G-hES cells had 2,230 genes up-regulated at day 6 of differentiation; Dox-treated G-hES cells had 2,417 genes that were up-regulated at day 6 differentiation; 1,332 genes were co-up-regulated by RA and GCNF (Fig. 5*E*). RA-treated G-hES cells had 2,062 genes down-regulated at day 6 of differentiation; Dox-treated G-hES cells had 2,400 genes down-regulated at day 6 of differentiation; and 1,302 genes were co-down-regulated by RA and GCNF at day 6 of differentiation (Fig. 5*F*). The altered genes with *p* < 0.001 are listed in supplemental Table S1. These data show that GCNF can regulate global gene expression both in undifferentiated hES cells and during differentiation of hES cells.

## Discussion

This study is the first to show that *GCNF* is a key transcription factor that induces differentiation of hES cells by repressing Oct4 expression. GCNF is expressed at very low levels in undifferentiated hES cells, but at a level insufficient to repress Oct4, and is transiently increased during RA-induced hES differentiation, which leads to the repression of Oct4. In contrast, in loss-of-function experiments Oct4 expression is maintained when GCNF expression is knocked down by siRNA, even in the presence of RA. These results were consistent with previous results reported in mouse pluripotent stem cells (15, 17–19). Previous studies have shown that the level of Oct4 expression is directly related to ES cell pluripotency and cell fate decisions (24). *Oct4* deficient knock-out embryos develop to the blastocyst stage, however the pluripotent ICM cells differentiate along the trophectoderm lineage, and no ICM cells are established (25). Similar results were obtained in early mouse development when the Oct4 gene was knocked-down using small interfering RNA methodology (26).

Our results show that the level of Oct4 expression depends on the level of GCNF expression. In the first gain-of-function experiments, over-expression of GCNF induces inhibition of Oct4 expression in undifferentiated hES cells, which results in spontaneous hES cell differentiation and changes in lineage commitment in differentiated hES cells (6, 27). According to a study in mES cells, a less than 2-fold increase from normal expression levels causes differentiation into ectoderm and mesoderm, whereas a reduction to less than 50% leads to the differentiation into trophectoderm (24). Knockdown of *Oct4* results in rapid differentiation of hES cells and a significant increase in transcription of genes associated with trophoblastic and endoderm lineages (27). During hES cell differentiation, the level of Oct4 expression decreases more quickly after Dox-induced GCNF expression plus RA treatment than after RA treatment alone. This indicates that GCNF enhances the repression of Oct4 during RA-induced hES cell differentiation. Similar results were seen in mouse embryonic studies. During gastrulation in mouse embryonic development, *GCNF* is expressed in ectodermal structures and the primitive streak of the embryo at E6.5. At E9.5, GCNF expression becomes more restricted to the developing nervous system and is drastically downregulated by E10.5. In parallel, Oct4 expression gradually decreases, whereas loss of GCNF expression in KO mouse models results in the sustained expression of Oct4 in somatic portions of the embryos where it is not normally expressed (19).

GCNF induces the repression of pluripotent genes that are expressed in undifferentiated hES cells that are normally down-regulated during differentiation. Induction of GCNF also leads to up-regulation of some genes in the undifferentiated state (Fig. 5). This is likely an indirect effect as GCNF is a transcriptional repressor with no known transactivation function (28). Compared with RA-induced differentiation of ES cells, GCNF overexpression is sufficient to downregulate pluripotent gene expression leading to differentiation of hES cells, which indicates that GCNF regulates gene expression directly and plays a crucial role in the differentiation process. In a related study, neuronal cells develop immaturely after GCNF was knocked out in ES cells, which eliminated repression of Oct4 expression (29). Oct4 is a master transcription factor that regulates the pluripotent gene circuitry that maintains the self-renewal and pluripotency of ES cells (30, 31). GCNF disrupts this regulatory circuitry by inhibiting expression of Oct4 and other pluripotent factors triggering in part hES cell differentiation. Identifying factors that inhibit expression of key transcription factors in hES cells is a critical step in understanding the maintenance of pluripotency and regulation of hES cell differentiation.

We have shown for the first time that *Oct4* expression is regulated by GCNF in a dose-dependent manner during hES cell differentiation. Decreasing the level of GCNF expression by siRNA leads to maintenance of Oct4 expression during hES cell differentiation; in contrast, in the first gain-of-function experiments overexpression of GCNF promotes repression of Oct4 expression, leading in turn to hES cell differentiation. Global analysis of gene expression in response to perturbation of GCNF expression shows that it regulates most of the pluripotency genes either directly or indirectly, regulating gene expression in undifferentiated hES cells when over expressed and during hES differentiation normally. As an orphan member of the nuclear receptor family of ligand-activated transcription factors our data establish a potential role for manipulating pluripotency through small molecule agonists or antagonists of GCNF, which has implications for regenerative medicine applications.

## Author Contributions

H. W. conducted most of the experiments, analyzed the results, and wrote most of the paper. X. W. conducted experiments on the roles GCNF overexpression repressing Oct4 in hES cells. X. X. conducted experiments on GCNF regulating global gene expression. M. K. conducted GCNF overexpression in hES cells. A. J. C. conceived the idea for the project, and wrote the paper with H. W.

## Supplementary Material

Supplemental Data

## Footnotes

3 The abbreviations used are:

ES: embryonic stem
ICM: inner cell mass
GCNF: germ cell nuclear factor
Dox: doxycycline
EMSA: electrophoretic mobility shift assay
ChIP: chromatin immunoprecipitation.
