# Supplementary material for: Germ Cell Nuclear Factor (GCNF) Represses Oct4 Expression and Globally Modulates Gene Expression in Human Embryonic Stem (hES) Cells
Source: J Biol Chem. 2016 Jan 14;291(16):8644–52. doi: 10.1074/jbc.M115.694208 (PMC4861434; doi:10.1074/jbc.M115.694208)
Supplement: Supplemental Data [file supp_291_16_8644__index.html]

Germ Cell Nuclear Factor (GCNF) Represses Oct4 Expression and Globally Modulates Gene Expression in Human Embryonic Stem (hES) Cells — Germ Cell Nuclear Factor (GCNF) Represses Oct4 Expression and Globally Modulates Gene Expression in Human Embryonic Stem (hES) Cells — GCNF Represses Oct4 in hES Cells — Supplemental Data 

# Germ Cell Nuclear Factor (GCNF) Represses Oct4 Expression and Globally Modulates Gene Expression in Human Embryonic Stem (hES) Cells

## Supplemental Data

- supplemental table (.xlsx, 16 KB) - This file is the altered genes in the analysis of mRNA microarray data
